# Learning Spoken Words via the Ears and Eyes: Evidence from 30-Month-Old Children

**DOI:** 10.3389/fpsyg.2017.02122

**Published:** 2017-12-08

**Authors:** Mélanie Havy, Pascal Zesiger

**Affiliations:** Faculty of Psychology and Educational Sciences, University of Geneva, Geneva, Switzerland

**Keywords:** audio-visual speech perception, word-learning, cross-modal recognition, lexical representation, child development

## Abstract

From the very first moments of their lives, infants are able to link specific movements of the visual articulators to auditory speech signals. However, recent evidence indicates that infants focus primarily on auditory speech signals when learning new words. Here, we ask whether 30-month-old children are able to learn new words based solely on visible speech information, and whether information from both auditory and visual modalities is available after learning in only one modality. To test this, children were taught new lexical mappings. One group of children experienced the words in the auditory modality (i.e., acoustic form of the word with no accompanying face). Another group experienced the words in the visual modality (seeing a silent talking face). Lexical recognition was tested in either the learning modality or in the other modality. Results revealed successful word learning in either modality. Results further showed cross-modal recognition following an auditory-only, but not a visual-only, experience of the words. Together, these findings suggest that visible speech becomes increasingly informative for the purpose of lexical learning, but that an auditory-only experience evokes a cross-modal representation of the words.

## Introduction

From the very first moments of their lives, infants experience the multisensory nature of speech. Through face-to-face interactions, infants simultaneously hear the auditory speech signal and see the accompanying movements of the speaker’s face (Altvater-Mackensen and Grossmann, 2015). In adults, visible speech conveys redundant and complementary information (Miller and Nicely, 1955; Robert-Ribes et al., 1998) that reliably enhances auditory phonetic perception (i.e., Samuel and Lieblich, 2014) and facilitates lexical recognition (Brancazio, 2004; Barutchu et al., 2008; Buchwald et al., 2009; Fort et al., 2010, 2013; Havy et al., 2017). Here we ask whether young children, who have considerably less experience in watching others’ articulators, can benefit from visible speech as they learn new words.

Studies with infants document a surprisingly early preparedness for audio-visual speech perception. As they enter the world, infants are sensitive to the dynamic properties of faces (Guellaï et al., 2011) and can link specific movements of the visual articulators to the auditory speech signal across different dimensions (temporal: Lewkowicz and Pons, 2013; Baart et al., 2014, spectral: Kuhl et al., 1991). For instance, when presented with a side-by-side display of two talking faces producing two different vowels, and using the auditory track of a single vowel, infants spontaneously look more at the face which matches the auditory track (i.e., by 2 months for vowels: Kuhl and Meltzoff, 1982; Patterson and Werker, 2003; Streri et al., 2016; by 6 months for consonants: Pons et al., 2009). Infants do not merely associate auditory and visible speech, but can integrate both sets of information. This can typically be seen in the McGurk effect, in which conflicting auditory (i.e., /ba/) and visible speech information (i.e., /ga/) of syllables lead to a unified percept, which integrates both sensory modalities (i.e., /da/) (i.e., at 2–5 months: Burnham and Dodd, 2004; Bristow et al., 2009).

Over the course of development, multisensory perceptual abilities become increasingly more sophisticated and greatly influence language acquisition. By 6–10 months, infants deploy selective attention to the mouth of a talking face (Lewkowicz and Hansen-Tift, 2012) and can discern one language from another simply from viewing the silently articulated face (Weikum et al., 2007; Lewkowicz and Pons, 2013; Kubicek et al., 2014). Around the same age, infants use visible speech to resolve the identity of individual phonemes and learn novel phonetic categories (Teinonen et al., 2008). As they accumulate language experience, infants retain the intersensory relations that are phonemically relevant to their native language (rhythmic: Lewkowicz and Pons, 2013; Kubicek et al., 2014; phonetic: Pons et al., 2009; Danielson et al., 2017). However, little is known about how, and how early in development, visible speech begins to shape lexical representations.

Given their initial predispositions, infants should be able to represent visible speech information as they learn their first words. This is supported by evidence linking early attentiveness to visible speech to later lexical achievements. In particular, infants who pay more attention to the mouth of their interlocutor (Young et al., 2009) and respond more to incongruent audio-visual vowels at 6 months (Altvater-Mackensen et al., 2016) are more likely to have higher receptive and expressive vocabularies at 12 (Altvater-Mackensen et al., 2016) and 24 months, respectively (Young et al., 2009).

Yet, alternative evidence paints a more nuanced picture and suggests instead a relative independence of visible speech from the lexical domain until late in development. First, studies have revealed that infants’ attention is not equal across auditory and visual modalities. When processing audio-visual compounds involving objects and non-speech sounds, infants exhibit an auditory dominance and allocate more attention to the auditory than to the visual information (i.e., at 8–16 months: Robinson and Sloutsky, 2004; Sloutsky and Robinson, 2008). This bias attenuates as they advance in age, especially during pre-school years (Robinson and Sloutsky, 2004).

Second, there is evidence from speech perception studies that audio-visual processing of non-sense forms (sounds, sound combinations, pseudo-words) continues to improve throughout childhood. For example, audio-visual matching is not consistently found for certain vowels until 9 months (Altvater-Mackensen et al., 2016; Streri et al., 2016) and for sine-wave speech until 6 years (Baart et al., 2015). Further, infants (i.e., at 4 months: Desjardins and Werker, 2004) and school-age children from three to 8 years old are less amenable to the McGurk illusion than older children (i.e., at 11 years) and adults and are more likely to have an auditory capture (i.e., /ba/) of the conflict (i.e., auditory /ba/ and visual /ga/; McGurk and MacDonald, 1976; Sekiyama and Burnham, 2008).

Third, the literature on familiar word recognition offers diverging evidence in childhood. For example, there is evidence that infants/children can differentiate words from pseudo-words in both the auditory and the visual modalities (i.e., at 12–13 months: Weatherhead and White, 2017) and can use visible speech to boost lexical recognition in normal (i.e., at four and 10–14 years: Jerger et al., 2009) and adverse listening conditions (i.e., at 3–4 years: Grieco-Calub and Olson, 2015; Lalonde and Holt, 2015; at 6–14 years: Ross et al., 2011; Maidment et al., 2014). Yet, contrasting evidence also reports that children do not reliably attend to visible speech. For example, Jerger et al. (2014) found that 4-year-old children were able to use the visual input to restore the excised onset of pseudo-words presented auditorily but did not show the visible speech fill-in effect for words (Jerger et al., 2014). In conjunction with this, Fort et al. (2012) found that 6-to-10-year-old children were able to identify the presence of a target phoneme within words and pseudo-words embedded in noise (Fort et al., 2012). Phoneme identification was higher in the audio-visual than in the auditory modality and overall was higher for words than for pseudo-words (word superiority effect). The word superiority effect was evident in the auditory and the audio-visual modalities. Yet, and unlike for adults, it was not increased by the presence of visible speech, as visible speech enhanced perception equally for words and pseudo words. This was interpreted as evidence that early on, visible speech contributes to pre-lexical units (sounds and sound combinations which do not take into consideration their associated meaning) more than to lexical units (sounds and sound combinations which take into consideration their associated meaning).

In general, the current literature does not provide any clear-cut evidence as to whether visible speech is contained in early lexical representations. Task demands may account for part of the observed variability, as a greater visual influence has been observed on indirect measures (implicit retrieval), which require less processing resources than direct measures (overt responses) (Jerger et al., 2009, 2014). Critically, much of the current research does not distinguish between the two mechanisms whereby visible speech may become part of lexical representations. First, visible speech may be stored directly by encoding the visually available information from the input. Second, visible speech may be incorporated through cross-modal translation of the auditory input. To what extent these mechanisms shape early lexical representations and their precise time course of development remains largely unknown.

However, one research project has begun to address this issue. In their research, Havy et al. (2017) asked whether 18-month-old English-learning infants were able to learn new lexical mappings in either auditory or visual modality (Havy et al., 2017). The purpose was twofold: firstly to determine whether visible speech alone can be used to guide lexical learning, and secondly whether information from either auditory or visual modalities is available through cross-modal translation of the input. The task involved learning the name of two objects (Word A-Object A and Word B-Object B) in either auditory (auditory stream only) or visual modality (silent talking face), while asked when tested to look at the object being named. Lexical recognition was tested either in the same modality as the one used during the learning phase (same modality test condition, i.e., auditory after auditory learning, visual after visual learning) or in the other modality (cross-modality test condition, i.e., visual after auditory learning, auditory after visual learning). The results revealed that infants were able to learn new word-referent mappings in the auditory modality and when tested could recognize the mapping when presented either in the auditory or visual modality. However, unlike adults, infants did not show evidence of lexical learning in the visual modality. This pattern was interpreted as evidence that at 18 months, infants favor auditory speech information as they learn new lexical mappings and represent visible speech through cross-modal translation of the auditory input. This finding is consistent with the view discussed earlier, suggesting a general auditory dominance in how infants process audio-visual information (Robinson and Sloutsky, 2004; Sloutsky and Robinson, 2008).

Following on from Havy et al. (2017), the purpose of the current study is to determine at what age children reliably start to use visible speech as they learn words. We have focused on the age of 30 months, as it corresponds to a precisely timed change in the maturation of several capacities relevant to audio-visual word learning. By this age, children show greater audio-visual sensitivities (Grieco-Calub and Olson, 2015), greater fast-mapping capacities (Vlach and Sandhofer, 2012; Wojcik, 2013), more detailed word form representations (Floccia et al., 2014) and higher receptive and expressive vocabularies (Ganger and Brent, 2004; McMurray, 2007). Building on the same design as the one used by Havy et al. (2017), we ask (1) whether 30-month-old children are able to establish new lexical representations based on visible speech information alone, and (2) whether information from either auditory or visual modalities can be part of representations through cross-modal translation of the input. We reason that general achievements in different aspects of perception, language, and cognition at 30 months may positively influence how children attend to the visible correlates of speech during word learning. Alternatively, it may be the case that children are still encountering obstacles in navigating across the auditory and visual modalities. If so, children may experience difficulties in learning words visually and/or difficulties in recovering the auditory correspondents of the words which have been learned visually.

## Materials and Methods

### Participants

Forty monolingual French-learning children participated in the study at the University of Geneva, **Table 1**. All the children were healthy, full-term and with no known developmental delay or history of vision or hearing impairments. The children came from families living in Geneva and were exposed to French for more than 80% of the time. All were recruited through birth records and enrolled with their parents’ consent in accordance with the recommendations of the Ethics Committee of the University of Geneva. The children’s families received a small gift by way of compensation. An additional 26 children were tested but excluded from the analysis due to excessive fussiness/crying (*n* = 11), failure to contribute to each test condition (*n* = 4), calibration issue (*n* = 3) or poor tracking ratio (*n* = 8). The tracking ratio was defined as the amount of time the eye-tracker recorded the gaze coordinates over the entire task and was deemed unreliable if lower than 30%. In the final sample, 21 children formed the auditory learning group (9 males, 12 females; *M* = 30 months, 26 days, *range:* 29 months, 9 days – 32 months, 19 days), **Table 1**. Another nineteen children formed the visual learning group (10 males, nine females; *M* = 30 months, 16 days, *range:* 28 months, 20 days – 32 months, 13 days), **Table 1**. Both groups came from middle-class socio-economic backgrounds [*t*(36) = 0.53, *p* = 0.63, *d* = 0.17; see Havy et al., 2016, for calculation of SES scores taking into account the professional and education achievement of both parents] and had comparable word-learning capacities [*t*(35) = 1.49, *p* = 0.15, *d* = 0.50; as assessed by the French version of the MacArthur-Bates Communicative Development Inventory for expressive vocabulary, Kern, 2003), **Table 1**.

**Table 1:** Demographic information including the participant’s identification (ID), the age (Months; Days), the gender (Male, Female), the MCDI estimation of the expressive vocabulary size and the socio-economic status (SES) as well as the mean and standard deviation (SD) for each group.

	Age (Months; Days)	Gender (Male, Female)	MCDI in production	SES score
**Auditory learning group**				

**Mean (*SD*)**	**30;26 (0;33)**	**9 Males**	**420 (114)**	**22.26 (4.13)**

ID				
1	29;28	Male	Unknown	28
2	30;01	Female	Unknown	21
3	30;04	Male	481	24
4	29;27	Female	567	13
5	30;00	Female	516	Unknown
6	30;10	Male	231	Unknown
7	30;02	Female	419	18
8	29;24	Female	499	26
9	31;18	Male	387	25
10	30;02	Male	Unknown	22
11	31;26	Female	287	27
12	30;18	Female	477	26
13	32;19	Male	353	20
14	32;09	Female	335	18
15	33;15	Male	602	25
16	31;09	Female	417	26
17	31;18	Female	499	20
18	31;19	Female	394	20
19	29;09	Male	511	22
20	30;26	Male	413	16
21	31;02	Female	169	26

**Visual learning group**				

**Mean (*SD*)**	**30;16 (0;34)**	**10 Males**	**361 (127)**	**21.32 (5.12)**

ID				
1	32;02	Female	476	26
2	29;01	Male	232	16
3	30;07	Female	425	22
4	29;05	Male	200	23
5	30;05	Female	326	28
6	29;29	Male	329	24
7	30;14	Female	159	16
8	29;11	Male	370	16
9	32;13	Male	552	26
10	28;20	Male	181	26
11	29;24	Female	396	26
12	32;05	Male	641	24
13	31;25	Male	398	23
14	31;28	Female	299	25
15	30;07	Female	270	18
16	30;27	Male	384	18
17	30;29	Female	351	15
18	29;25	Male	522	24
19	31;01	Female	342	9

### Stimuli

#### Speech Stimuli

The speech stimuli consisted of four pairs of French-sounding words with a CVC structure (consonant – vowel – consonant: /*byp*/-/*var*/, /*rik*/-/*fal*/, /*fyf*/-/*gel*/, /*mum*/-/*tit*/). The pseudo-words were selected to afford maximum distinctiveness in both the auditory and visual modalities (see Binnie et al., 1974; Jackson et al., 1976 for phonetic confusion matrices). These contrasted by at least two features in each segment (manner, place and voicing for the consonants, backness, height and roundness for the vowels) and could be easily discriminated by 30-month-old children (Kuhl and Meltzoff, 1982; Patterson and Werker, 2003; Desjardins and Werker, 2004; Werker and Curtin, 2005; Pons et al., 2009; Yeung and Werker, 2013; Tsuji and Cristia, 2014; Streri et al., 2016).

The pseudo-words were recorded by a native French female speaker in a child-friendly form of directed speech. The pseudo-words were produced in a carrier phrase: determiner (‘un’) + pseudo-word, so as to highlight the referential status to the word (Fennell and Waxman, 2010) and to ensure that children began attending to the speech information prior to the word onset. Three tokens of each pseudo-word were selected and matched for duration and intonation contour. Two of them (two for each pseudo-word) were used for the familiarization, learning and test phases and another one (one for each word) was used solely for the learning and test phases.

All speech stimuli were recorded using a Sony HDR-CX730 video camera at 50 frames per second. Digital capture and editing were done using Audacity (Audacity, version 2.0.5) and Final Cut Pro (Final Cut Pro, version 7.0.3). For each word, three media sequences were created: an audio-visual sequence, an auditory-only sequence (sound stream with the video stream removed), and a visual-only sequence (video stream with the sound stream removed). The mean duration of audio-visual sequences was 1208 ms (*SD* = 58 ms, *range* = 1125–1291 ms). The auditory stream was edited to ensure similar sound levels across the stimuli (60 dB). The video sequences were cropped to remove all external features above the hairline and below the neck of the talking face. The background detail of the talking face was replaced by a uniform light gray background. The videos were positioned at the center of the screen, with a display size of approximately 16° × 16° of visual angle at a viewing distance of 60 cm.

#### Object Stimuli

**FIGURE 1:**
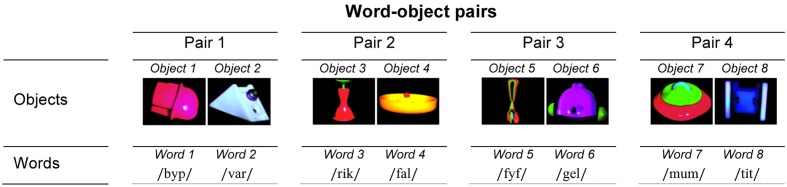
The four word-object pairs used. The word-object association is counterbalanced across participants.

Four pairs of novel objects were created using Photoshop (Adobe Photoshop CS4, version 11.0) and Final Cut Pro (Final Cut Pro, version 7.0.3). All objects were colorful and had similar levels of detail. Within each pair, the two objects differed by about 55.86% of their RGB value, brightness and shape [pair 1: 58.08%, pair 2: 45.92%, pair 3: 52.04%, pair 4: 67.40% (Resemblejs, version 2.2.0)] and were easily discriminable. To sustain the children’s visual interest, each object was presented against a black background and rotated along a vertical axis for 2750 ms (Johnson, 2010). The objects were presented alone at the center of the screen for the learning phase and together, side by side, for the test phase. From a viewing distance of 60 cm, the objects subtended approximately 16° × 16° of the visual angle for the learning phase and 10° × 10° for the test phase. There was a gap of about six visual degrees between the objects when tested. Each pair of objects was randomly assigned to a unique pair of pseudo-words (pair 1: objects 1 and 2 with pseudo-words 1 and 2; pair 2: objects 3 and 4 with pseudo-words 3 and 4; pair 3: objects 5 and 6 with pseudo-words 5 and 6; pair 4: objects 7 and 8 with pseudo-words 7 and 8, **Figure 1**). A smooth, undulating shape with a display size of 3° × 3° of the visual angle was used as an attention-getter.

### Apparatus

The visual stimuli were presented on a 22-inch Dell E2209W monitor with a resolution of 1680 pixels × 1050 pixels per inch and a refresh rate of 60 Hz. The auditory stimuli were presented through left-right loudspeakers at a conversational level. Calibration procedure and stimuli presentations were run using a Dell Latitude E6520 laptop. Data were monitored using I-view (I-view, version 2.8.26) and Experiment Center (Experiment Center, version 3.2.17) native to SMI (SensoMotoric Instruments GmbH, Teltow, Germany). The children’s eye-movements were recorded by means of a SMI RED500 eye-tracking device with a sampling rate of 60 Hz.

### Procedure

The study was conducted in a dimly lit, sound-attenuated room at the University of Geneva. The children were seated on a caregiver’s lap at a viewing distance of approximately 60 cm from the eye-tracker monitor set-up. The caregiver was instructed to keep their eyes closed, and not to talk, point to the screen or influence the child’s attention. The session was initiated by a five-point calibration routine, where a spinning wheel was shown individually at five points on the screen: one at each corner and one at the center. The calibration procedure was gaze-contingent and was repeated as necessary.

**FIGURE 2:**
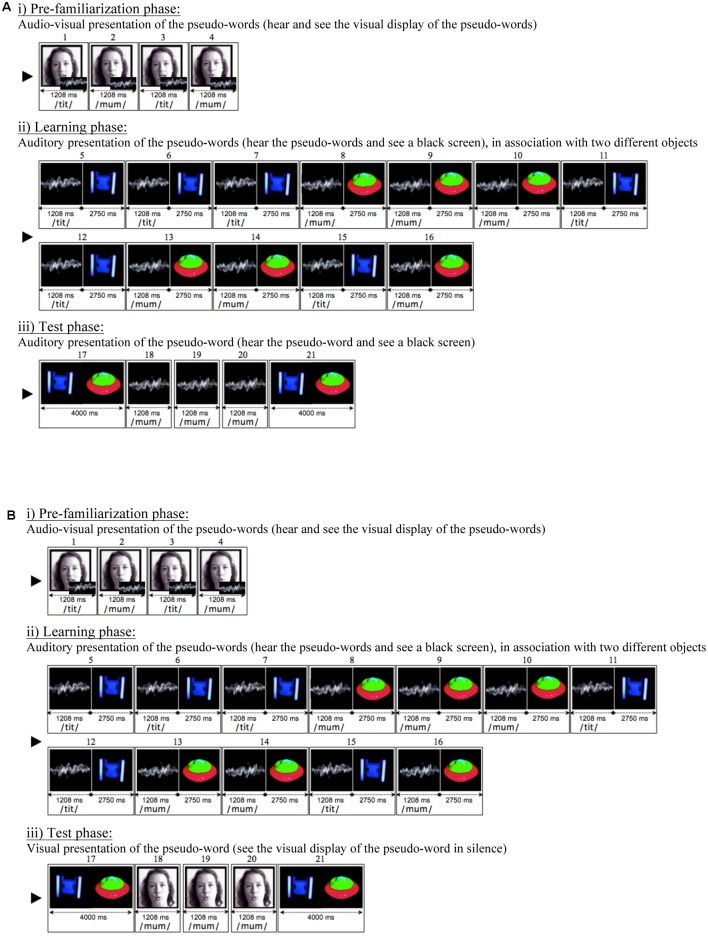
Schematic of a ‘same modality’ **(A)** and a ‘cross-modality’ **(B)** test trial in the auditory word-learning condition. The sequences of events are depicted in their actual order (from 1 to 21) for each experimental test trial. The sine wave represents the sound heard and is not actually seen on the screen.

Following a successful calibration, the children were given four experimental trials. Each trial consisted of three phases: (i) a pre-familiarization phase, (ii) a learning phase and (iii) a test phase (**Figure 2**). The children completed the three phases of one trial before moving to the next trial.

(i) During the pre-familiarization phase, the children were introduced to two pseudo-words in their audio-visual mode (seeing and hearing a talking face). This audio-visual presentation of the pseudo-words was intended to attract the children’s attention to the multisensory aspect of speech. This pre-familiarization potentially provided specific information that could facilitate learning and support cross-modal recognition of the target words. However, there was no evidence of such an effect in Havy et al. (2017), as the infants failed to learn in the visual modality. Besides, care was taken to insure that the children would not consider the pre-familiarization as a hint for cross-modal recognition. The pre-familiarization was kept to a minimum, and even shortened in relation to the original design (Havy et al., 2017). This was motivated by evidence from categorization studies, which showed that cross-modal influences of auditory speech information on visual object processing are only evident after a certain amount of trials (Althaus and Plunkett, 2016). Here, the pseudo-words were uttered only twice in alternation, with a different realization each time (pseudo-word 1: token 1, pseudo-word 2: token 1, pseudo-word 1: token 2, pseudo-word 2: token 2). A variability in realization was expected to help infants identify the relevant contrastive dimension of the words being learned (Rost and McMurray, 2009).

(ii) Immediately after the pre-familiarization, the children entered a lexical learning phase. During the lexical learning, the same two pseudo-words were presented in association with two distinct objects. One group of children experienced the words in the auditory modality (hearing the sound but seeing a black screen, **Figure 2**). Another group experienced the words in the visual modality (seeing the silent talking face). Each word immediately preceded the object’s appearance. To foster learning and highlight the lexically relevant phonological information, the learning phase contained three different tokens of each pseudo-word which were played twice (two tokens from the pre-familiarization phase and a novel one). The object pairs were arranged in the following order: three iterations of one pair followed by three iterations of the other, two iterations of the first pair followed by two iterations of the other and, lastly one iteration of each pair. This order was intended to engage the infants’ selective attention on one object at a time and thus facilitate learning. Details of the learning phase progression are as follows: pseudo-word 1: token 1 – object 1, pseudo-word 1: token 2 – object 1, pseudo-word 1: token 3 – object 1, pseudo-word 2: token 1 – object 2, pseudo-word 2: token 2 – object 2, pseudo-word 2: token 3 – object 2, pseudo-word 1: token 1 – object 1, pseudo-word 1: token 2 – object 1, pseudo-word 2: token 1 – object 2, pseudo-word 2: token 2 – object 2, pseudo-word 1: token 3 – object 1, pseudo-word 2: token 3 – object 2, with a 1 s interval between each pair. During the interval, and in order to sustain attention throughout the learning phase, children saw a smooth, undulating shape at the center of the screen.

(iii) Immediately after the learning phase, the test phase was initiated. The test phase began with the presentation of the two previously seen objects, side by side and in silence, for 4 s. (pre-naming period). Next, both objects disappeared and one of them was labeled three times, each time with a different realization (pseudo-word 1: token 1, pseudo-word 1: token 2, pseudo-word 1: token 3). After labeling, both objects reappeared side by side in silence for 4 s (post-naming period). The modality of labeling varied depending on the test condition. In the ‘same modality’ test condition, labeling occurred in the same modality as the one used at learning: i.e., auditory after auditory learning (hearing the pseudo-word and seeing a black screen, **Figure 2A**), and visual after visual learning (seeing the silent talking face). In the ‘cross-modality’ test condition, labeling occurred in the other modality to the one used at learning: i.e., visual after auditory learning (**Figure 2B**); and auditory after visual learning. Each trial tested one test condition only: either the ‘same modality’ or the ‘cross-modality’ condition. Out of the four experimental trials children received during the session, two of them tested the ‘same modality’ condition and the other two the ‘cross-modality’ condition. Sessions lasted approximately 20 min.

### Counterbalancing

Participants of each learning group were assigned randomly to one out of three protocols. Protocols were made up by varying the order of presentation of each word-object pair. For each word-object pair, the modality of labeling during the test phase (‘same modality’ vs. ‘cross-modality’) and which of the two objects was labeled (object A vs. object B) were balanced. All protocols started with a ‘same modality’ test trial to facilitate the children’s understanding of the task, followed by three other trials testing either condition (‘same modality’ vs. ‘cross-modality’) in a different order.

## Results

### Data Analyzes

Analyzes were performed using BeGaze (BeGaze, version 3.2.28). Data were analyzed with respect to two areas of interest (AOI): one corresponding to the location of the target object, the other corresponding to the location of the distractor. AOIs were defined by dividing the entire screen into two equal parts. We chose large AOIs to adjust for variations in object size across trials and minimize artifacts in how BeGaze interpolates eye position (de Urabain et al., 2015; Hessels et al., 2016). Gaze data consisted of the sum of durations for all saccades and fixations hitting the AOIs. In line with Havy et al. (2017), we considered for analysis the 4-s periods before (pre-naming period) and after naming (post-naming period) and calculated for each period the proportion of target-looking time, that is, the amount of time spent looking at the target object (T) over the amount of time spent looking at the target (T) and the distractor object (D): T/(T + D).

### Data Cleaning

Data cleaning consisted of a series of six filters successively applied to the initial dataset. The dataset initially included 176 trials for 44 participants, and upon filtering consisted of 137 trials for 40 participants (auditory learning: 68/84 trials; visual learning: 69/76 trials). Filters were applied in line with Havy et al. (2017). We first trimmed ten trials from the initial dataset in which the children were not fixating on the monitor during the familiarization and learning phases (Filter 1: auditory learning: 6/88 trials; visual learning: 4/88 trials). We then removed six visual test trials (auditory learning: cross-modality; visual learning: same modality) in which the children were not looking at the model during the visual-only naming period of the test phase (Filter 2: auditory learning: 4/44 trials; visual learning: 2/44 trials). Based on criteria commonly used in two-choice word learning and word recognition tasks (Swingley and Aslin, 2000; Havy et al., 2017), we discarded 11 additional trials in which children were not fixating on the monitor during the pre-naming and/or post-naming periods of the test phase (Filter 3: auditory learning: 7/88 trials; visual learning: 4/88 trials). We then controlled for biases in spontaneous object preferences and identified three trials in which children attended to either one of the objects during the pre-naming period (auditory learning: 2/88; visual learning: 1/88). These trials were included in the analyses, as the bias did not last throughout the post-naming period (Filter 4, Delle Luche et al., 2015). On the remaining 149 trials, we screened for atypical data points falling outside normality. We considered data points larger than 2 SD from the mean as outliers (Fernald et al., 2006) and disregarded four additional trials (Filter 5: auditory learning: 1/88 trials; visual learning 3/88 trials). The exclusion of these four trials resulted in the exclusion of the four corresponding participants, who no longer contributed to the experimental conditions (Filter 6: auditory learning: *n* = 1; visual learning *n* = 3; Fernald et al., 2006). The exclusion of these four participants resulted in the removal of eight trials (Filter 6: auditory learning: 2/88 trials; visual learning: 6/88 trials). A total of 39 trials (auditory learning: 20/88 trials; visual learning: 19/88 trials) were thus removed from the original dataset.

### Mixed Effects Model

Statistical analyzes were performed using SPSS (SPSS, version 21). To assess the contribution of learning and test conditions to the children’ performance, we ran a linear mixed effects model, using the percentage of target-looking time per trial and per child as a dependent measure. As fixed effects, we entered the learning condition, the test condition and the naming period in the model, as well as all interactions between these three predictors. The learning condition referred to the modality of labeling at learning (‘auditory’ vs. ‘visual’). The test condition referred to the modality of labeling during the test, namely the same modality as the one used during learning (‘same modality’ test) or the other modality (‘cross-modality’ test). The naming period corresponded to the period of time prior to labeling (‘pre-naming’ period) and after labeling (“post-naming” period) of one of the two objects. The random part of the model initially included random intercepts for participants and items and random slopes which allowed for the differing effects of the naming period and the test conditions across participants. The inclusion of random slopes typically corrects type 1 error rates and ensures that the results are not driven by a restricted set of participants or items (Baayen et al., 2008; Jaeger, 2008). However, these terms were subsequently removed due to a lack of convergence. The results reported here stem from an intercept-only model. The model was applied using a maximum-likelihood estimation. Estimates, standard errors and *t*-values are reported with *t* > 2 being interpreted as significant. *T*-tests were also performed to compare the mean proportion of looking times (averaged over the trials for each condition) against chance (set at 50%, since each response involved a choice between two equally probable possibilities).

The results of the mixed effects model yielded a significant main effect of the naming period [β = 13.69%, *SE* = 4.70%, *t*(223.94) = 2.91, *p* < 0.01]; a significant naming period^∗^ test condition interaction [β = -15.65%, *SE* = 6.69%, *t*(223.94) = 2.34, *p* = 0.02]; and a significant naming period^∗^ learning^∗^ test condition interaction [β = 19.51%, *SE* = 9.50%, *t*(223.94) = 2.05, *p* = 0.04], **Table 2**.

**Table 2:** Table showing the results of a maximum-likelihood estimated model predicting infants’ performance.

Parameters		Parameters estimates		
		Mean (*SD*)	Estimate (*SE*)	*T*-test statistics
**FIXED EFFECTS**				
Main effects and interactions				
Learning		–	4.02 (7.49)	*t*(18.10) = 0.54, *p* = 0.59
Test condition		–	7.80 (8.09)	*t*(14.12) = 0.97, *p* = 0.35
**Naming period**		–	13.69 (4.70)	***t*(223.94) = 2.91, *p* < 0.01**
Test period^∗^ learning		–	2.85 (6.69)	*t*(223.94) = 0.43, *p* = 0.67
**Naming period^∗^ test condition**		–	-15.65 (6.69)	***t*(223.94) = 2.34, *p* = 0.02**
Learning^∗^ test condition		–	-9.97 (10.49)	*t*(17.64) = 0.95, *p* = 0.36
**Naming period^∗^ learning^∗^ test condition**		–	19.51 (9.50)	***t*(223.94) = 2.05, *p* = 0.04**

			**Variance (*SE*)**	**Wald *Z* statistics**

**RANDOM EFFECTS**				
Subjects on intercepts		–	0.13 (0.17)	*Wald Z* = 0.78, *p* = 0.44
Items on intercepts		–	0.43 (0.25)	*Wald Z* = 1.74, *p* = 0.08

		**Mean (*SD*)**	**Estimate (*SE*)**	***T*-test statistics**

**PLANNED COMPARISONS**				
Auditory				
**Overall**	Pre-naming	49.67 (15.81)	18.47 (3.39)	***t*(105.33) = 5.44, *p* < 0.01**
	Post-naming	68.98 (18.29)		
**Same modality**	Pre-naming	49.69 (15.60)	16.54 (4.63)	***t*(44.34) = 3.57, *p* < 0.01**
	Post-naming	68.24 (19.33)		
**Cross-modality**	Pre-naming	49.64 (16.39)	20.40 (4.73)	***t*(46.80) = 4.31, *p* < 0.01**
	Post-naming	69.71 (17.62)		
Visual				
Overall	Pre-naming	49.25 (11.03)	5.98 (3.39)	*t*(133.98) = 1.76, *p* = 0.08
	Post-naming	53.93 (19.64)		
**Same modality**	Pre-naming	46.42 (9.63)	13.69 (4.44)	***t*(67.99) = 3.08, *p* < 0.01**
	Post-naming	59.97 (13.26)		
Cross-modality	Pre-naming	52.08 (11.95)	-1.95 (4.96)	*t*(65.99) = 0.39, *p* = 0.69
	Post-naming	47.89 (23.24)		

*Parameter estimates include the mean performance (Mean) in the different conditions, the estimated coefficient (Estimate) of the fixed effects and *t*-test statistics, the Variance of the random effects and Wald *Z* statistics. Planned comparisons test naming effects (naming period: pre-naming vs. post-naming) in each learning (auditory vs. visual) and test (same modality vs. cross-modality) condition. Significant main effects and interactions are in bold*

**FIGURE 3:**
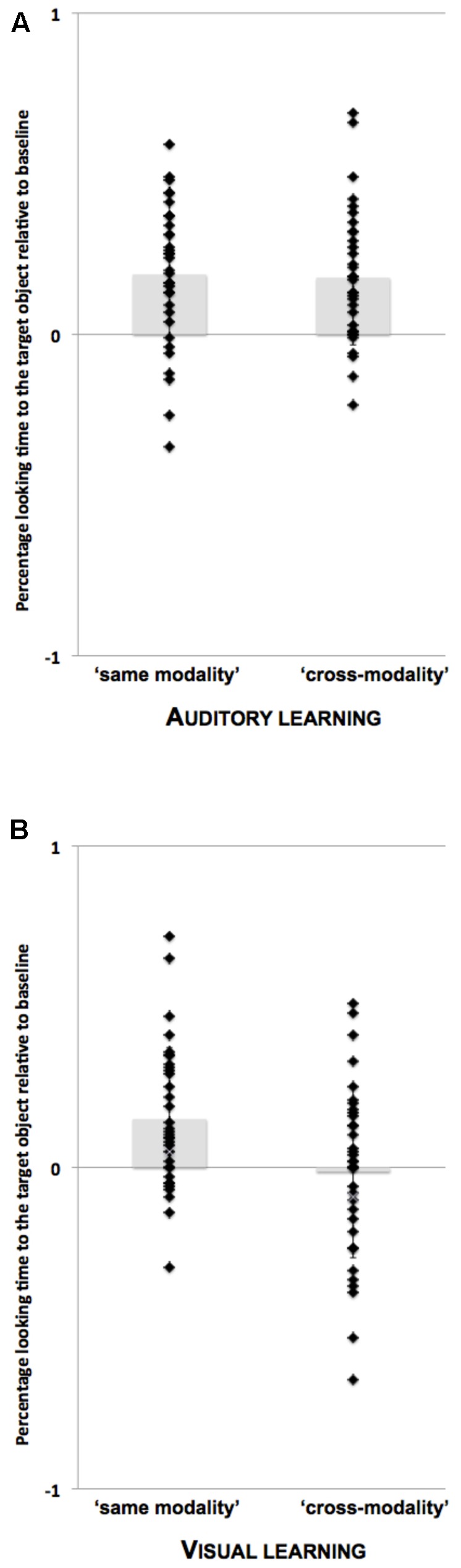
Mean proportion of target-looking times [Target/(Target + Distractor)] after adjusting for baseline preferences (post-naming period minus pre-naming period). Positive values indicate greater looking at the target object rather than the distractor upon naming. Individual data points are overlaid on group means for the auditory learning group **(A)** and the visual learning group **(B)** in the two ‘same modality’ and the two ‘cross-modality’ test trials. Each participant contributes to at least one data point in each test condition (‘same modality’ vs. ‘cross-modality’). This makes a total of 66 observations for the auditory learning group (same modality: 34/42, cross-modality: 34/42) and 69 observations for the visual learning group (same modality: 35/38, cross-modality: 34/38). Error bars represent the standard deviation from the mean.

Decomposition of the interactions revealed differences between the auditory and visual learning groups (see **Figure 3** and **Table 2**). The children in the auditory learning group demonstrated a reliable increase in target-looking preference after presentation of the word in both the ‘same modality’ [β = 16.54%, *SE* = 4.63%, *t*(44.34) = 3.57, *p* < 0.01]; and ‘cross-modality’ test conditions [β = 20.40%, *SE* = 4.73%, *t*(46.80) = 4.31, *p* < 0.01]. There was no looking preference for either object before naming: ‘same modality’ (*M* = 49.69%, *SD* = 15.60%), *t* < 1; ‘cross-modality’ (*M* = 49.64%, *SD* = 16.39%), *t* < 1; but a significant preference for the target object after naming: ‘same modality’ (*M* = 68.24%, *SD* = 19.33%), *t*(20) = 4.32, *p* < 0.001, *d* = 0.93; ‘cross-modality’ (*M* = 69.71%, *SD* = 17.62%), *t*(20) = 5.13, *p* < 0.001, *d* = 1.01.

The children in the visual learning group were just as likely to identify the target object upon naming, but they did so only in the ‘same modality’ test condition: ‘same modality’ [β = 13.69%, *SE* = 4.44%, *t*(67.99) = 3.08, *p* < 0.01]; ‘cross-modality’ [β = -1.95%, *SE* = 4.96%, *t*(65.99) = 0.39, *p* = 0.69]. There was no preference for either object before naming: ‘same modality’ (*M* = 46.42%, *SD* = 9.63%), *t*(18) = 1.62*, p* = 0.12, *d* = 0.60; cross-modality (*M* = 52.08%, *SD* = 11.85%), *t* < 1; but a significant preference for the target object after naming in the ‘same modality’ condition only: ‘same modality’ (*M* = 59.97%, *SD* = 13.26%), *t*(18) = 3.28, *p* = 0.004, *d* = 0.85; ‘cross-modality’ (*M* = 47.89%, *SD* = 23.24%), *t* < 1.

Other effects and interactions did not reach significance (All *t*s < 2). Wald *Z* statistics revealed that the variation in the participants’ (*Wald Z* = 0.78, *p* = 0.44) and items’ (*Wald Z* = 1.74, *p* = 0.08) intercepts was not confounded by our effects of primary theoretical interest.

## Discussion

The purpose of the current study was to identify two mechanisms whereby visible speech might become part of lexical representations. First, we asked whether 30-month-old children are able to use visible speech alone to guide lexical learning. Secondly, we examined whether information from either auditory or visual modalities could be part of new lexical representations through cross-modal translation of the input. To test this, we used the same word-learning task as in Havy et al. (2017). Children were taught new lexical mappings in either auditory or visual modalities, then tested for recognition either in the same modality as at learning (‘same modality’ test condition) or in the other modality (‘cross-modality’ test condition).

First, our results revealed that 30-month-old children are able to form new lexical mappings after a short auditory exposure to the word forms. This pattern is consistent with previous evidence documenting early auditory word-learning capacities in different laboratory settings (Werker and Curtin, 2005; Havy and Nazzi, 2009; Vouloumanos and Werker, 2009; Yoshida et al., 2009; Havy et al., 2017). Of primary interest, our results further revealed that 30-month-old children are able to learn new lexical mappings solely based on visible speech information. This finding is in line with Havy et al. (2017), which shows that in adults, visible speech may be stored directly from the input without support from the auditory domain. Critically, the current finding, in conjunction with Havy et al. (2017), revealed a transition in the development of this mechanism. In particular, it showed that, unlike 18-month-olds who primarily attend to the auditory speech signal, 30-month-olds exploit either source of information. This suggests that visible speech becomes increasingly informative for lexical learning.

With regard to our second question, our results demonstrated that visible speech can be part of lexical representations, even after an auditory-only experience of the words. This pattern fits well with Havy et al. (2017) which shows cross-modal translation of the auditory input at 18 months and in adults. However, unlike in adults, auditory recognition was not found for visually learned words. This suggests that despite greater sensitivities to the visible correlates of speech, representations of visually learned words are still not adult like.

To summarize, the current evidence underscores the existence at 30 months of two individual mechanisms for encoding visible speech into the lexicon: one building directly on the visually available information and another building on cross-modal translation of the auditory input. This work also highlights an important milestone in how children appreciate visible speech information. While younger children incorporate visible speech into the lexicon only indirectly, older ones use both a direct and an indirect processing route. This distinction, which highlights the contribution of visible speech, also raises important questions about what could influence the observed developmental change.

Firstly, it is possible that the observed change could be attributed to a general improvement in audio-visual speech perception. This is supported by accumulated evidence showing greater responsiveness to visible speech information in normal and adverse listening conditions (Ross et al., 2011) and greater visual capture of the McGurk effect over the course of development (McGurk and MacDonald, 1976; Desjardins and Werker, 2004; Sekiyama and Burnham, 2008).

However, broader perceptual reorganizations occur concurrently that may also influence performance. During the 2nd year of life, children show an increased interest in a broad range of visual stimuli (i.e., objects: Bornstein and Arterberry, 2010; Zamuner et al., 2014; faces: Frank et al., 2014). This greater precision in processing visual information may also promote the use of visible speech. Yet, initial interest in visual information is not the same in all situations and is substantially higher when visual input is accompanied by speech sounds (Waxman and Braun, 2005; Ferry et al., 2010) or non-speech sounds that are given a clear communicative function (tones: Ferguson and Waxman, 2016, conspecific calls: Perszyk and Waxman, 2016) than non-speech sounds that have no linguistic interpretation (Robinson and Sloutsky, 2004). This suggests that multisensory associations are constrained with regards to the linguistic nature of the information under consideration, which in turn challenges the possibility that the observed shift is linked to domain-general mechanisms.

This leads us to move beyond considerations of perceptual sensitivities alone and to identify what could possibly influence the use of visible speech in the lexical domain. Several achievements are noteworthy. One of them concerns the overall attention devoted to the word form. Early on, infants possess remarkable discrimination capacities. However, there is considerable variability in how they appreciate the auditory detail of word forms as they associate words with objects (Werker et al., 2002; Havy and Nazzi, 2009). As they advance in age, children construct more accurate phonetic representations of words (Floccia et al., 2014). These capacities, which have been documented for the most part in the auditory speech domain, may also extend to the visible speech domain. Future studies will aim to test this possibility by considering how children visually treat words that are minimally distinct.

Another possibility is that children form different expectations about the type of signal that can be accepted as a word. For example, there is evidence that by 30 months, children are less likely to accept manual gestures as a word (Sheehan et al., 2007; Suanda et al., 2013). This decline in receptivity to gestural labels, in conjunction with our finding of an increased sensitivity to visible speech, suggest a different selectivity for the type of visual signals that are primarily linked to the lexicon. Future research will have to test this possibility by comparing how children learn from visible speech and manual gestures in the same design.

Finally, children’s emerging ability to produce words may change the emphasis and weight given to visible speech. During the 2nd year of life, lexical production dramatically increases and becomes more accurate (Fenson et al., 1994; Ganger and Brent, 2004). Yet, studies document that infants (Young et al., 2009; Yeung and Werker, 2013; Altvater-Mackensen and Grossmann, 2015; Streri et al., 2016) and children (Desjardins et al., 1997) are more sensitive to the visible speech information they can appropriately produce. This functional link with production and its feed-back to perception may influence children’s selective attention to visible speech. Future research will be aimed at exploring visual word learning when articulators are temporally restrained, or more permanently impaired, as in individuals with cleft palates.

In total, the current work demonstrates that children attend to visible speech more reliably as they establish new lexical representations. But it is important to note that, unlike adults, they show cross-modal recognition in the auditory modality only. This indicates that the representations/mechanisms involved during auditory and visual learning may be substantially different. What could explain such differences? In the literature, there is evidence that auditory speech and visible speech provide different amounts of information about the identity of the signal. While auditory speech has only one visual correspondence, visible speech can be matched with more than one auditory template. This uncertainty in the auditory translation of the visual input may place a particular demand on children who have to keep several alternative possibilities in their memories. Future research will need to evaluate this claim by manipulating the number of auditory candidates for the same visual input.

In line with the above, it is useful to consider the methodological limitations of the task. In everyday life, it is very common to hear a sound without seeing the corresponding articulatory movement, yet it is much less common to watch a silent face. In keeping with this, children in the current task noticed the absence of sound but did not react to the absence of a face. The lack of naturalness of the visual learning condition may be detrimental to performance, especially at a younger age. One direction for future studies could be to modify this condition by simultaneously playing the corresponding auditory signal at a low signal-to-noise ratio.

As part of the methodological limitations, it is also important to note the influence of the pre-familiarization phase. Prior to lexical learning, children were briefly introduced to the audio-visual form of the words. This pre-familiarization may have fostered lexical learning and assisted in the establishment of a recoverable cross-modal representation. But the audio-visual experience was short and there was no evidence of cross-modal recognition upon visual learning. Besides, the literature concurrently reports that audio-visual experience with a word may either facilitate or hinder auditory and visual recognition of this word when learning word-object associations in adults (Bernstein et al., 2013; Eberhardt et al., 2014). It is therefore unclear as to whether and how the multisensory information available during pre-familiarization influences lexical learning at 30 months and, if so, whether this effect changes over the course of development. Future studies will need to investigate the role of the pre-familiarization phase in the current task, possibly by excluding this phase or by testing a condition using different words during pre-familiarization and lexical learning.

Along with this methodological limitation, it is important to note the very limited number of pseudo-words that has been considered. Future studies will need to replicate this work whilst using a broader set of pseudo-words. Language-specific effects may have also influenced the current finding. Previous evidence documents various sensitivities to the McGurk illusion depending on the language being used (Sekiyama and Burnham, 2008). Future studies will need to investigate how the sensory format of lexical representations develops across languages.

Another avenue for future research will be to determine the nature and number of representations that are at play. One possibility is that, at 30 months, children form two modality-specific representations: one based on the available input and another that is uniquely activated through cross-modal translation of the auditory input. If so, differences in performance between the auditory and visual learning conditions may reflect differences in the cross-modal transfer mechanisms, either in terms of presence/absence of the mechanisms or in terms of relative robustness. The visual-to-auditory transfer may emerge later than the auditory-to-visual transfer or be already present but not reliably used. Another possibility is that children form one single representation. This means that after auditory learning, the representation that is formed is cross-modal and contains information from both sensory modalities. After visual learning, the representation may be cross-modal or sensory-specified. If cross-modal, the observed differences may reflect differences in the robustness and confidence associated with the representations. If sensory-specified, the observed differences may reflect differences in the sensory format of the representations, from sensory-specified to cross-modal. It is also important to note that the nature and the number of representations involved may be susceptible to developmental change. Children may move from two separate representations to a more abstract cross-modal representation as they gain more language experience.

## Conclusion

The current study reveals the contribution of visible speech in 30-month-old lexical representations. It provides evidence of two mechanisms for encoding visible speech, each associated with a different timing of emergence. Futures studies will need to explore more closely the developmental transition observed between 18 and 30 months and how cross-linguistic differences in attending to visible speech influence the sensory format of early lexical representations.

## Ethics Statement

This study was carried out in accordance with the recommendations of the ethic committee of Geneva University with written informed consent from all subjects. All subjects gave written informed consent in accordance with the Declaration of Helsinki. The protocol was approved by the ethic committee of Geneva University.

## Author Contributions

MH designed the study, tested the participants and wrote the paper. PZ provided insightful comments that improved the quality of the manuscript.

## Conflict of Interest Statement

The authors declare that the research was conducted in the absence of any commercial or financial relationships that could be construed as a potential conflict of interest.

## References

[B1] Althaus N. Plunkett K. (2016). Categorization in infancy: labeling induces a persisting focus on commonalities. *Dev. Sci.* 19 770–780. 10.1111/desc.12358 26538010 PMC4995729

[B2] Altvater-Mackensen N. Grossmann T. (2015). Learning to match auditory and visual speech cues: social influences on acquisition of phonological categories. *Child Dev.* 86 362–378. 10.1111/cdev.12320 25403424

[B3] Altvater-Mackensen N. Mani N. Grossmann T. (2016). Audiovisual speech perception in infancy: the influence of vowel identity and infants’ productive abilities on sensitivity to (mis)matches between auditory and visual speech cues. *Dev. Psychol.* 52 191–204. 10.1037/a0039964 26595352

[B4] Baart M. Bortfeld H. Vroomen J. (2015). Phonetic matching of auditory and visual speech develops during childhood: evidence from sine-wave speech. *J. Exp. Child Psychol.* 129 157–164. 10.1016/j.jecp.2014.08.002 25258018 PMC4252499

[B5] Baart M. Vroomen J. Shaw K. Bortfeld H. (2014). Degrading phonetic information affects matching of audiovisual speech in adults, but not in infants. *Cognition* 130 31–43. 10.1016/j.cognition.2013.09.006 24141035 PMC3904288

[B6] Baayen R. H. Davidson D. J. Bates D. M. (2008). Mixed-effects modeling with crossed random effects for subjects and items. *J. Mem. Lang.* 59 390–412. 10.1016/j.jml.2007.12.005

[B7] Barutchu A. Crewther S. G. Kiely P. Murphy M. J. Crewther D. P. (2008). When /b/ill with /g/ill becomes /d/ill: Evidence for a lexical effect in audiovisual speech perception. *Eur. J. Cogn. Psychol.* 20 1–11. 10.1016/j.jecp.2014.05.003 24974346 PMC4106987

[B8] Bernstein L. E. Auer E. T. Jr. Eberhardt S. P. Jiang J. (2013). Auditory perceptual learning for speech perception can be enhanced by audiovisual training. *Front. Neurosci.* 7:34 10.3389/fnins.2013.00034 PMC3600826 23515520

[B9] Binnie C. A. Montgomery A. A. Jackson P. L. (1974). Auditory and visual contributions to the perception of consonants. *J. Speech Lang. Hear. Res.* 17 619–630. 10.1044/jshr.1704.619 4444283

[B10] Bornstein M. H. Arterberry M. E. (2010). The development of object categorization in young children: hierarchical inclusiveness, age, perceptual attribute, and group versus individual analyses. *Dev. Psychol.* 46 350–365. 10.1037/a0018411 20210495 PMC2856652

[B11] Brancazio L. (2004). Lexical influences in audiovisual speech perception. *J. Exp. Psychol. Hum. Percept. Perform.* 30 445–463. 10.1037/0096-1523.30.3.445 15161378

[B12] Bristow D. Dehaene-Lambertz G. Mattout J. Soares C. Gliga T. Baillet S. (2009). Hearing faces: how the infant brain matches the face it sees with the speech it hears. *J. Cogn. Neurosci.* 21 905–921. 10.1162/jocn.2009.21076 18702595

[B13] Buchwald A. Winters S. J. Pisoni D. B. (2009). Visual speech primes open-set recognition of auditory words. *Lang. Cogn. Process.* 24 580–610. 10.1080/01690960802536357 21544260 PMC3085279

[B14] Burnham D. Dodd B. (2004). Auditory-visual speech integration by prelinguistic infants: perception of an emergent consonant in the McGurk effect. *Dev. Psychobiol.* 45 204–220. 10.1002/dev.20032 15549685

[B15] Danielson D. K. Bruderer A. G. Kandhadai P. Vatikiotis-Bateson E. Werker J. F. (2017). The organization and reorganization of audiovisual speech perception in the first year of life. *Cogn. Dev.* 42 37–48. 10.1016/j.cogdev.2017.02.004 28970650 PMC5621752

[B16] de Urabain I. R. S. Johnson M. H. Smith T. J. (2015). GraFIX: a semiautomatic approach for parsing low-and high-quality eye-tracking data. *Behav. Res. Methods* 47 53–72. 10.3758/s13428-014-0456-0 24671827 PMC4333362

[B17] Delle Luche C. Durrant S. Poltrock S. Floccia C. (2015). A methodological investigation of the intermodal preferential looking paradigm: methods of analyses, picture selection and data rejection criteria. *Infant Behav. Dev.* 40 151–172. 10.1016/j.infbeh.2015.05.005 26176183

[B18] Desjardins R. Werker J. F. (2004). Is the integration of heard and seen speech mandatory for infants? *Dev. Psychobiol.* 45 187–203. 10.1002/dev.20033 15549681

[B19] Desjardins R. N. Rogers J. Werker J. F. (1997). An exploration of why preschoolers perform differently than do adults in audiovisual speech perception tasks. *J. Exp. Child Psychol.* 66 85–110. 9226935 10.1006/jecp.1997.2379

[B20] Eberhardt S. P. Auer E. T. Jr. Bernstein L. E. (2014). Multisensory training can promote or impede visual perceptual learning of speech stimuli: visual-tactile vs. visual-auditory training. *Front. Hum. Neurosci.* 8:829. 10.3389/fnhum.2014.00829 25400566 PMC4215828

[B21] Fennell C. T. Waxman S. R. (2010). What paradox? Referential cues allow for infant use of phonetic detail in word learning. *Child Dev.* 81 1376–1383. 10.1111/j.1467-8624.2010.01479.x 20840228 PMC2941229

[B22] Fenson L. Dale P. S. Reznick J. S. Bates E. Thal D. (1994). Variability in early communicative development. *Monogr. Soc. Res. Child Dev.* 59 1–173; discussion174–185. 7845413

[B23] Ferguson B. Waxman S. R. (2016). What the [beep]? Six-month-olds link novel communicative signals to meaning. *Cognition* 146 185–189. 10.1016/j.cognition.2015.09.020 26433024 PMC5347446

[B24] Fernald A. Perfors A. Marchman V. A. (2006). Picking up speed in understanding: speech processing efficiency and vocabulary growth across the 2nd year. *Dev. Psychol.* 42 98–116. 10.1037/0012-1649.42.1.98 16420121 PMC3214591

[B25] Ferry A. Hespos S. J. Waxman S. (2010). Language facilitates category formation in 3-month-old infants. *Child Dev.* 81 472–479. 10.1111/j.1467-8624.2009.01408.x 20438453 PMC2910389

[B26] Floccia C. Nazzi T. Delle Luche C. Poltrock S. Goslin J. (2014). English-learning one-to two-year-olds do not show a consonant bias in word learning. *J. Child Lang.* 41 1085–1114. 10.1017/S0305000913000287 23866758

[B27] Fort M. Kandel S. Chipot J. Savariaux C. Granjon L. Spinelli E. (2013). Seeing the initial articulatory gestures of a word triggers lexical access. *Lang. Cogn. Process.* 28 1207–1223. 10.1080/01690965.2012.701758

[B28] Fort M. Spinelli E. Savariaux C. Kandel S. (2010). The word superiority effect in audiovisual speech perception. *Speech Commun.* 52 525–532. 10.1016/S0167-6393(98)00051-X

[B29] Fort M. Spinelli E. Savariaux C. Kandel S. (2012). Audiovisual vowel monitoring and the word superiority effect in children. *Int. J. Behav. Dev.* 36 457–467. 10.1177/0165025412447752

[B30] Frank M. C. Amso D. Johnson S. P. (2014). Visual search and attention to faces during early infancy. *J. Exp. Child Psychol.* 118 13–26. 10.1016/j.jecp.2013.08.012 24211654 PMC3844087

[B31] Ganger J. Brent M. R. (2004). Reexamining the vocabulary spurt. *Dev. Psychol.* 40 621–632. 10.1037/0012-1649.40.4.621 15238048

[B32] Grieco-Calub T. M. Olson J. (2015). Individual differences in real-time processing of audiovisual speech by preschool children. *J. Acoust. Soc. Am.* 137 2375–2375. 10.1121/1.4920629

[B33] Guellaï B. Coulon M. Streri A. (2011). The role of motion and speech in face recognition at birth. *Vis. Cogn.* 19 1212–1233. 10.1080/13506285.2011

[B34] Havy M. Bouchon C. Nazzi T. (2016). Phonetic processing when learning words: the case of bilingual infants. *Int. J. Behav. Dev.* 40 41–52. 10.1177/0165025415570646 20121879

[B35] Havy M. Foroud A. Fais L. Werker J. F. (2017). The role of auditory and visual speech in word-learning at 18 months and in adulthood. *Child Dev.* 88 2043–2059. 10.1111/cdev.12715 28124795 PMC6540983

[B36] Havy M. Nazzi T. (2009). Better processing of consonantal over vocalic information in word learning at 16 months of age. *Infancy* 14 439–456. 10.1080/15250000902996532 32693448

[B37] Hessels R. S. Kemner C. Van den Boomen C. Hooge I. T. (2016). The area-of-interest problem in eyetracking research: a noise-robust solution for face and sparse stimuli. *Behav. Res. Methods* 48 1694–1712. 10.3758/s13428-015-0676-y 26563395 PMC5101255

[B38] Jackson P. L. Montgomery A. A. Binnie C. A. (1976). Perceptual dimensions underlying vowel lipreading performance. *J. Speech Lang. Hear. Res.* 19 796–812. 1003957 10.1044/jshr.1904.796

[B39] Jaeger T. F. (2008). Categorical data analysis: away from ANOVAs (transformation or not) and towards logit mixed models. *J. Mem. Lang.* 59 434–446. 10.1016/j.jml.2007.11.007 19884961 PMC2613284

[B40] Jerger S. Damian M. F. Spence M. J. Tye-Murray N. Abdi H. (2009). Developmental shifts in children’s sensitivity to visual speech: a new multimodal picture–word task. *J. Exp. Child Psychol.* 102 40–59. 10.1016/j.jecp.2008.08.002 18829049 PMC2612128

[B41] Jerger S. Damian M. F. Tye-Murray N. Abdi H. (2014). Children use visual speech to compensate for non-intact auditory speech. *J. Exp. Child Psychol.* 126 295–312. 10.1016/j.jecp.2014.05.003 24974346 PMC4106987

[B42] Johnson S. P. (2010). How infants learn about the visual world. *Cogn. Sci.* 34 1158–1184. 10.1111/j.1551-6709.2010.01127.x 21116440 PMC2992385

[B43] Kern S. (2003). Le compte-rendu parental au service de l’évaluation de la production lexicale des enfants français entre 16 et 30 mois. *Glossa* 85 48–62.

[B44] Kubicek C. Gervain J. de Boisferon A. H. Pascalis O. Lœvenbruck H. Schwarzer G. (2014). The influence of infant-directed speech on 12-month-olds’ intersensory perception of fluent speech. *Infant Behav. Dev.* 37 644–651. 10.1016/j.infbeh.2014.08.010 25238663

[B45] Kuhl P. K. Meltzoff A. (1982). The bimodal perception of speech in infancy. *Science* 218 1138–1141. 7146899 10.1126/science.7146899

[B46] Kuhl P. K. Williams K. A. Meltzoff A. N. (1991). Cross-modal speech perception in adults and infants using nonspeech auditory stimuli. *J. Exp. Psychol. Hum. Percept. Perform.* 17 829–840. 1834794 10.1037//0096-1523.17.3.829

[B47] Lalonde K. Holt R. F. (2015). Preschoolers benefit from visually salient speech cues. *J. Speech Lang. Hear. Res.* 58 135–150. 10.1044/2014_JSLHR-H-13-0343 25322336 PMC4712850

[B48] Lewkowicz D. J. Hansen-Tift A. M. (2012). Infants deploy selective attention to the mouth of a talking face when learning speech. *Proc. Natl. Acad. Sci. U.S.A.* 109 1431–1436. 10.1073/pnas.1114783109 22307596 PMC3277111

[B49] Lewkowicz D. J. Pons F. (2013). Recognition of amodal language identity emerges in infancy. *Int. J. Behav. Dev.* 37 90–94. 10.1177/0165025412467582 24648601 PMC3956126

[B50] Maidment D. W. Kang H. J. Stewart H. J. Amitay S. (2014). Audiovisual integration in children listening to spectrally degraded speech. *J. Speech Lang. Hear. Res.* 58 61–68. 10.1044/2014_JSLHR-S-14-0044 25203539

[B51] McGurk H. MacDonald J. (1976). Hearing lips and seeing voices. *Nature* 264 746–748. 1012311 10.1038/264746a0

[B52] McMurray B. (2007). Defusing the childhood vocabulary explosion. *Science* 317:631. 10.1126/science.1144073 17673655

[B53] Miller G. A. Nicely P. E. (1955). An analysis of perceptual confusions among some English consonants. *J. Acoust. Soc. Am.* 27 338–346.

[B54] Patterson M. L. Werker J. F. (2003). Two-month-old infants match phonetic information in lips and voice. *Dev. Sci.* 6 191–196. 10.1111/1467-7687.00271

[B55] Perszyk D. Waxman S. R. (2016). Listening to the calls of the wild: the role of experience in linking language and cognition in young infants. *Cognition* 153 175–181. 10.1016/j.cognition.2016.05.004 27209387 PMC5134735

[B56] Pons F. Lewkowicz D. J. Soto-Faraco S. Sebastián-Gallés N. (2009). Narrowing of intersensory speech perception in infancy. *Proc. Natl. Acad. Sci. U.S.A.* 106 10598–10602. 10.1073/pnas.0904134106 19541648 PMC2705579

[B57] Robert-Ribes J. Schwartz J.-L. Lallouache T. Escudier P. (1998). Complementary and synergy in bimodal speech: auditory, visual and audiovisual identification of French oral vowels. *J. Acoust. Soc. Am.* 103 3677–3689. 9637049 10.1121/1.423069

[B58] Robinson C. W. Sloutsky V. M. (2004). Auditory dominance and its change in the course of development. *Child Dev.* 75 1387–1401. 10.1111/j.1467-8624.2004.00747.x 15369521

[B59] Ross L. A. Molholm S. Blanco D. Gomez-Ramirez M. Saint-Amour D. Foxe J. J. (2011). The development of multisensory speech perception continues into the late childhood years. *Eur. J. Neurosci.* 33 2329–2337. 10.1111/j.1460-9568.2011.07685.x 21615556 PMC3127459

[B60] Rost G. C. McMurray B. (2009). Speaker variability augments phonological processing in early word learning. *Dev. Sci.* 12 339–349. 10.1111/j.1467-7687.2008.00786.x 19143806 PMC3011987

[B61] Samuel A. G. Lieblich J. (2014). Visual speech acts differently than lexical context in supporting speech perception. *J. Exp. Psychol. Hum. Percept. Perform.* 40 1479–1490. 10.1037/a0036656 24749935 PMC4122614

[B62] Sekiyama K. Burnham D. (2008). Impact of language on development of auditory-visual speech perception. *Dev. Sci.* 11 306–320. 10.1111/j.1467-7687.2008.00677.x 18333984

[B63] Sheehan E. A. Namy L. L. Mills D. L. (2007). Developmental changes in neural activity to familiar words and gestures. *Brain Lang.* 101 246–259. 10.1016/j.bandl.2006.11.008 17250885

[B64] Sloutsky V. M. Robinson C. W. (2008). The role of words and sounds in infants’ visual processing: from overshadowing to attentional tuning. *Cogn. Sci.* 32 342–365. 10.1080/03640210701863495 21635339

[B65] Streri A. Coulon M. Marie J. Yeung H. H. (2016). Developmental change in infants’ detection of visual faces that match auditory vowels. *Infancy* 21 177–198. 10.1111/infa.12104

[B66] Suanda S. H. Walton K. M. Broesch T. Kolkin L. Namy L. L. (2013). Why two-year-olds fail to learn gestures as object labels: evidence from looking time and forced-choice measures. *Lang. Learn. Dev.* 9 50–65. 10.1080/15475441.2012.723189

[B67] Swingley D. Aslin R. N. (2000). Spoken word recognition and lexical representation in very young children. *Cognition* 76 147–166. 10856741 10.1016/s0010-0277(00)00081-0

[B68] Teinonen T. Aslin R. N. Alku P. Csibra G. (2008). Visual speech contributes to phonetic learning in 6-month-old infants. *Cognition* 108 850–855. 10.1016/j.cognition.2008.05.009 18590910

[B69] Tsuji S. Cristia A. (2014). Perceptual attunement in vowels: a meta-analysis. *Dev. Psychobiol.* 56 179–191. 10.1002/dev.21179 24273029

[B70] Vlach H. Sandhofer C. M. (2012). Fast mapping across time: memory processes support children’s retention of learned words. *Front. Psychol.* 3:46. 10.3389/fpsyg.2012.00046 22375132 PMC3286766

[B71] Vouloumanos A. Werker J. F. (2009). Infants’ learning of novel words in a stochastic environment. *Dev. Psychol.* 45 1611–1617. 10.1037/a0016134 19899918

[B72] Waxman S. R. Braun I. (2005). Consistent (but not variable) names as invitations to form object categories: new evidence from 12-month-old infants. *Cognition* 95 B59–B68. 10.1016/j.cognition.2004.09.003 15788158

[B73] Weatherhead D. White K. S. (2017). Read my lips: visual speech influences word processing in infants. *Cognition* 160 103–109. 10.1016/j.cognition.2017.01.002 28088039

[B74] Weikum W. M. Vouloumanos A. Navarra J. Soto-Faraco S. Sebastián-Gallés N. Werker J. F. (2007). Visual language discrimination in infancy. *Science* 316 1159–1159. 10.1126/science.1137686 17525331

[B75] Werker J. F. Curtin S. (2005). PRIMIR: A developmental framework of infant speech processing. *Lang. Learn. Dev.* 1 197–234. 10.1207/s15473341lld0102_4

[B76] Werker J. F. Fennell C. T. Corcoran K. M. Stager C. L. (2002). Infants’ ability to learn phonetically similar words: effects of age and vocabulary size. *Infancy* 3 1–30. 10.1207/S15327078IN0301_1

[B77] Wojcik E. H. (2013). Remembering new words: integrating early memory development into word learning. *Front. Psychol.* 4:151. 10.3389/fpsyg.2013.00151 23554599 PMC3612698

[B78] Yeung H. H. Werker J. F. (2013). Lip movements affect infants’ audiovisual speech perception. *Psychol. Sci.* 24 603–612. 10.1177/0956797612458802 23538910

[B79] Yoshida K. A. Fennell C. T. Swingley D. Werker J. F. (2009). Fourteen-month-old infants learn similar sounding words. *Dev. Sci.* 12 412–418. 10.1111/j.1467-7687.2008.00789.x 19371365 PMC2883913

[B80] Young G. S. Merin N. Rogers S. J. Ozonoff S. (2009). Gaze behavior and affect at 6 months: predicting clinical outcomes and language development in typically developing infants and infants at risk for autism. *Dev. Sci.* 12 798–814. 10.1111/j.1467-7687.2009.00833.x 19702771 PMC2732664

[B81] Zamuner T. S. Fais L. Werker J. F. (2014). Infants track word forms in early word–object associations. *Dev. Sci.* 17 481–491. 10.1111/desc.12149 24576138

